# National Education the Basis of National Health

**Published:** 1855-06

**Authors:** 


### NATIONAL EDUCATION THE BASIS OF NATIONAL HEALTH.

THE suggestion in a succeeding page, from the pen of the Rev. W. H. Lyttelton, on the teaching of sanitary knowledge to the children of the poor in the national schools of Great Britain, demands the especial attention of all those who are interested in the advance of sanitary science. Mr. Lyttelton, in his paper, has shown an intimate knowledge of the true state of the sanitary question, and has pointed out the only solid basis of national health. It is interesting, too, to see such thoughts emanating from a member of a profession which of all has most power to enlighten and influence the minds of the people. This is no time for any class of men, however firmly their position may be laid in antiquity, to rest inactive, or to act only as the dispensers of old established principles. If the clergy are to continue in vigorous life, they also must be up and doing; moving on in the true spirit of the age, grasping eagerly the scientific and general truths which the age is daily bringing forth, and teaching ever, by example as by precept, that to live behind the time is virtually to die.

At one period in the world's history, it was thought no trifling honour for high functionaries to combine in their own persons the duties of the priest and the physician. This combination is now broken, but the intimate relationship between physical and moral health remains as ever; and although it would be inadvisable, if not impossible under existing circumstances, to re-establish an obsolete system, still the duties of the clergyman and the physician should always go hand in hand; nor is the former ever performing more exalted functions than when teaching in simple language the physical nature of life, the agencies by which it is maintained, the influence of the healthy body in developing the healthy mind, and all the exquisite truths connected with the great laws of the Great Lawgiver.

Such instructions, indeed, form in themselves a kind of religion; they generate a love for truth, an intimacy with, and as a result, a firm confidence in the immutable ordinances of nature, and a correct appreciation of the facts and details of daily life.

With these feelings, it is incumbent on us to direct the attention of the clergy to the great importance of Mr. Lyttelton's proposition. Superintending, as they do at this moment, the educational system of this great country, which has intrusted to their hands the mental cultivation of the coming generation, it behoves them to review with great care their responsible position, and to recognise the necessity of taking broad views on educational subjects.

When the mind begins to consider the question of teaching sanitary lessons to the child population, many new and important suggestions soon present themselves. Simple models, showing the construction of cottages, public buildings, plans for ventilation, drainage, and the like, might at little cost be introduced into our schools, and soon become as familiar to the eye as maps and globes; even the grammar book might well find a friendly rival in the lesson book on health.

At this moment a government, however well-disposed to the task, could not, if it tried, institute a health code fully suited to the requirements of the time. Why is this? Simply because deep-rooted prejudices, self-interests based only on narrow views and really leading to self-injury, stand in the way. To remove these obstacles there is but one resource; namely, to prevent their introduction into the clear and docile mind of the learning child. A far-seeing government, anxious for the health and prosperity of its people, may, perhaps, some day recognise these facts, base many national blessings upon them, and immortalise itself.

